# Blending integrated knowledge translation with global health governance: an approach for advancing action on a wicked problem

**DOI:** 10.1186/s12961-019-0424-3

**Published:** 2019-03-04

**Authors:** Katrina Marie Plamondon, Julia Pemberton

**Affiliations:** 1 University of British Columbia, 3333 University Way, Kelowna, BC Canada; 2 McMaster University, 1280 Main Street West, Hamilton, ON Canada; 3 Interior Health, 505 Doyle Avenue, Kelowna, BC Canada

**Keywords:** Integrated knowledge translation, global health governance, health equity, health inequities, knowledge-to-action, wicked problems, complexity

## Abstract

**Background:**

The persistence of health inequities is a wicked problem for which there is strong evidence of causal roots in the maldistribution of power, resources and money within and between countries. Though the evidence is clear, the solutions are far from straightforward. Integrated knowledge translation (IKT) ought to be well suited for designing evidence-informed solutions, yet current frameworks are limited in their capacity to navigate complexity. Global health governance (GHG) also ought to be well suited to advance action, but a lack of accountability, inclusion and integration of evidence gives rise to politically driven action. Recognising a persistent struggle for meaningful action, we invite contemplation about how blending IKT with GHG could leverage the strengths of both processes to advance health equity.

**Discussion:**

Action on root causes of health inequities implicates disruption of structures and systems that shape how society is organised. This infinitely complex work demands sophisticated examination of drivers and disrupters of inequities and a vast imagination for who (and what) should be engaged. Yet, underlying tendencies toward reductionism seem to drive superficial responses. Where IKT models lack consideration of issues of power and provide little direction for how to support cohesive efforts toward a common goal, recent calls from the field of GHG may provide insight into these issues. Additionally, though GHG is criticised for its lack of attention to using evidence, IKT offers approaches and strategies for collaborative processes of generating and refining knowledge. Contemplating the inclusion of governance in IKT requires re-examining roles, responsibilities, power and voice in processes of connecting knowledge with action. We argue for expanding IKT models to include GHG as a means of considering the complexity of issues and opening new possibilities for evidence-informed action on wicked problems.

**Conclusion:**

Integrated learning between these two fields, adopting principles of GHG alongside the strategies of IKT, is a promising opportunity to strengthen leadership for health equity action.

## Background

Problems described as ‘wicked’ earn the label from their inherent resistance to resolve; they are convoluted, reactive problems entangled in competing social interests and values [1, 2]. The persistence of health inequities [3, 4] is a wicked problem [5] shaped by systems of power [5–10] and the social and structural determinants of health [11–13]. Robust evidence provides clear insights into their socio-political, economic and historic causes [14], and offers actionable policy solutions [15–18], making the persistence of health inequities a knowledge-to-action problem. However, efforts to advance evidence-informed action unfold in the same systems of social and political power that disproportionately advantage the already privileged and are generative of health inequities’ wicked nature. Additionally, despite recognition of their wickedness, attempts to respond often reduce health inequities into component parts, examining ‘symptoms’ rather than causes [19–21] in ways that ‘fit’ with dominant political ideologies [22–24]. These factors fuel the wickedness and tenacity of health inequities.

The purpose of this review article is to explore the complementarities of two promising approaches of applied research and practice that might support meaningful processes for advancing evidence-informed health equity action. It began with informal conversation about our experiences as doctoral students doing research for health equity, where we found ourselves grappling with contradictions and tensions within our field. Though we witnessed a broad commitment to ‘good’ equity intentions, we simultaneously observed processes and leadership that contradicted the evidence on causes and applauded superficial responses to health inequities. Critically examining how to move beyond good intentions for health equity became central to both of us in our doctoral research, with Katrina focusing on integrated knowledge translation (IKT) and Julia on global health governance (GHG). As our dialogue became more purposeful and structured, we discovered that the challenges we encountered in our respective fields were met by strengths in the other. Adopting an intentionally optimistic lens, we explored how these fields might complement each other and, through deeper attentiveness to issues of political power, could collectively contribute to more productive health equity action.

We understand both IKT and GHG to be primarily concerned with processes. IKT brings together people who do and use research as equal contributors to processes of collaboratively identifying and responding to knowledge-to-action challenges [25–28]. Ideally, GHG brings cross-sector actors together to deliberate and guide mechanisms for resolving complex global issues through intentional collaboration [29, 30]. Both are promising, yet the strengths of each mirror weaknesses in the other. Poor governance suffers from accountability and administrative failures, and a lack of strategy for integrating evidence-informed, contextual and tacit knowledges [31–33]. Emerging from health systems settings with clearly defined and contained contexts (e.g. clinical practice sites), IKT suffers a lack of attention to power dynamics and complexity in decision-making [27], making it difficult to apply to ‘wicked’ knowledge-to-action problems. Further, despite much interest in both IKT and GHG within the field of health equity, their respective bodies of literature are disconnected.

In this article, we draw on Freire and Shor’s metaphor of a ‘dialogic table’ [34], inviting contemplation of how blending IKT approaches with GHG principles could support meaningful health equity action. Below, we lay a foundation for deeper, critically reflective consideration of the complementarities of IKT and GHG. We are inspired by the potential of critical pedagogy as an epistemological guide for ‘how’ we (society, scientists, practitioners, decision-makers, etc.) might collectively inspire transformative possibilities. In their reflective conversation about learning, Freire and Shor described a ‘dialogic table’ as an enabler of transformative co-learning. They suggested an “*object to be known is put on the table between subjects…*[who] *meet around it and through it for mutual inquiry*” ([34], p. 14). The “*object to be known*” in this dialogue is evidence-informed health equity action. The ‘subjects of knowing’, each with their own insights, knowledges (e.g. tacit, institutional, professional knowledges), evidences and epistemologies, are people situated within the fields of IKT and GHG. We set this dialogic table by discussing the wickedness of health inequities, the strengths and limitations of IKT and GHG, and how to leverage their mutually beneficial characteristics.

## Unpacking the wickedness of health inequities

Health inequities are systematic differences in health rooted in socioeconomic and political injustices [14]. The WHO Commission on Social Determinants of Health described health inequities as avoidable, arising from “*…the conditions of daily life in which people are born, grow, live, work, and age*” shaped by social, political and economic forces, and requiring response from the “*whole of government*” [14]. Evidence shows that the greater the gap between a population’s richest and poorest, the greater the differences in health between them [3, 35, 36]. Unequal and unfair systems of power between and within nation states are widely recognised as driving forces in the creation of structures that disproportionately advantage some lives at the cost of others [8, 14, 37]. Though the evidence about causal roots is clear, and a robust suite of tested policy recommendations widely available [15–18], the implementation of policy solutions is far from straightforward. Calls for social policy reform to improve health have been documented for more than 160 years [38, 39], revealing deep resistance to resolve. Indeed, the causes of health inequities are tenacious because they are rooted socio-political systems and structures designed to reinforce the status quo of power distribution, locally and globally.

Connecting knowledge to action on root causes of health inequities implicates a reconstruction of the systems and structures that shape how society is organised. This infinitely complex work demands sophisticated examination of drivers and disrupters of inequities and a vast imagination for who (and what) should be engaged. This work is challenging for many reasons, not the least of which is a fundamental clash between health inequities’ inherent complexity and the dominant lens through which the world is observed and responded to in the field of health and science generally. This lens involves linear, reductionist and hierarchical assumptions (Table 1) stemming from seventeenth-century mechanistic suppositions about reality [20]. A repercussion of these assumptions is a persistent Western habit of understanding “*the world as a collection of separable and thus independent units and assumes linear cause-and-effect relationships between these units, and that these relationships are reversible*” ([21], p. 3). When these assumptions are at play, our collective capacity to recognise the depth and tenacity of root causes remains elusive.

**Table 1:** Mechanistic assumptions and their application to social determinants of health^a^

Assumption	Description	How the assumption circumvents complexity of health inequities
Reductionism	Assumes the whole system can be understood by identifying, describing and analysing all of its constituent parts	Breaks social determinants of health into separate, distinct factors (rather than a set of complex intersecting factors) Draws attention to symptoms or expressions of root causes that are more immediately visible (e.g. considering ‘race’ a determinant of health instead of ‘racism’)
Linearity	Assumes that (1) output changes proportionally with input, and (2) the effect of combined inputs can be understood and predicted by dissecting the input–output relationships of individual components, or a direct summative and predictive cumulation of constituent parts	Simplifies interconnectedness Justifies use of proxy indicators that reflect symptoms rather than causes of health inequities (e.g. monitoring maternal and child mortality rates as indicators of equity)
Hierarchy	Assumes central power and control, which diffuses systematically from proximal to more distal parts	Places responsibility for acting on health within individuals or groups, rather than society Legitimises a focus on health damaging behaviours rather than health damaging conditions, systems or structures

^a^Adapted from Jayasinghe, 2011 [20]

Lending to a particularly narrowed and superficial lens through which the social determinants of health [20] and health inequities are framed [24, 40], mechanistic assumptions effectively mask complex mechanisms that entrench inequities. Rather than focusing on the intersecting nature of the social determination of health [41], efforts to advance health equity under these assumptions place inordinate attention on behavioural interventions and insufficient attention on structural causes [42, 43]. For example, even when there is agreement about causes, public health efforts tend to focus on interventions that place responsibility for health on individual behaviours [23, 44]. Despite the recognised value of upstream and structural interventions, research shows a predominantly downstream focus in policy and public health efforts [24, 45–48]. Behavioural interventions for healthy eating, for example, distract attention away from complex issues of affordability and accessibility, whereas a more structural intervention might involve advocacy to advance socially protective policy for living wages.

Further, the role of power in establishing systematic advantage and disadvantage, recognised as a pivotal driver of health inequities [14], is only occasionally acknowledged and infrequently used to guide study goals and objectives [40]. Decades of dominant neoliberal ideology [47] have contributed to policy environments incompatible with the kinds of social protection known to mitigate health inequities [47, 49, 50]. Compounding these incompatibilities is a preoccupation with individualism and bio-behaviourism in health sciences that conflicts with the best available evidence and often distracts attention from where it might be most productive [24, 40, 51, 52]. Whether inadvertent or strategic, the absence of power analysis in efforts to advance health equity action can undermine possibilities of uprooting the tenacious systems of power that lead to inequities.

The fields of GHG and IKT span practice, policy and research outside the confines of a particular topic. Both fields bring something important to the table in response to health inequities. Further, because of their relational nature, they both offer platforms for the kind of dialogue necessary to challenge reductionism and mitigate power imbalances. Greater integration across disciplines interested in health equity is recognised as necessary evidence-informed action for health equity [53, 54]. If there is indeed desire and capacity to begin unravelling equity-harming structures, power and policy environments, then there is an urgent need to understand how to mobilise knowledge into action – both in terms of increasing the application of existing knowledge and informing emerging research. Unpacking these influences could provide a useful means of deconstructing underlying assumptions that lend themselves to consistent failures to advance health equity.

## What does IKT bring to the table?

Efforts to respond to health inequities include explicit calls for connecting research to action [14, 55]. These calls align with the growing recognition of the importance of knowledge translation (KT) [56–59]. IKT offers strategies for bringing diverse perspectives together to understand and respond to problems through processes of knowledge generation and refinement [25–27]. Inherently relational [60], IKT is non-linear and challenges traditional notions of the dispassionate, objective ‘expert researcher’ [61] whose work, once released into the world through scholarly publication, carries de facto impact. It involves participatory, inclusive processes where people who ‘use’ research work alongside people who ‘do’ research [62]. Recognising a ‘social contract’ between society and science, IKT brings stakeholders into a social process of problem solving through research [63] emphasising knowledge co-production in partnership [27]. By virtue of this collaborative approach to knowledge production, refinement and use, an IKT approach necessitates dialogue and trust building [64–66]. These characteristics are well suited to overcoming mechanistic assumptions by fostering ‘change from within’; however, the application of IKT to wicked problems is constrained by underlying assumptions that limit the scope and scale of contexts for which it was originally envisioned.

Frameworks for IKT consistently describe it as a way of collaboratively leveraging the research processes as a means for generating context-sensitive, complexity-embracing, real-life solutions grounded in evidence. Among evolving models for IKT are encouraging innovations, such as the use of critical realism and arts in KT [67], systems thinking [68], and even reflexive frameworks for equity-focused KT [69]. Common among these models is a recognition that “*both communities* [of knowledge users and producers] *hold distinct norms and values but they also bring valuable knowledge to the problem; and the work of knowledge generation is done collaboratively*” ([27], p. 620). A distinguishing feature of IKT is, however, that “*knowledge users usually have the authority to invoke change in the practice or policy setting*” [27]. This presumes that knowledge users are individual ‘stakeholders’ who represent particular portfolios within a health system or community setting. When the context and knowledge-to-action problem implicates social organisation and structure, however, the idea of including everyone, or even of finding just one set of stakeholders who may have authority to invoke change over some aspect of policy or practice relevant to health equity can be paralysing. The need for engaging people who can be part of decision-making mechanisms that lead to action opens a question of governance.

Although IKT models demonstrate promise for micro- (e.g. clinical practice unit) and meso- (e.g. health systems) contexts [70], their utility is limited when applied to the multiple, complex actors that contribute to shaping political, social and cultural environments that either drive, do nothing or disrupt wicked problems like health inequities. This is, in part, due to the difficulty of navigating meaningful engagement within the vastness of potential actors to include. Rather than focusing stakeholder analysis [71] in a defined setting, the range of potential actors implicated in wicked problems extends to networks of knowledge producers and knowledge users, many of which are not single entities, but conglomerates that also produce multiple competing interests and values. Identifying the ‘right’ actors to engage could become in and of itself a wicked problem, resistant to resolve and surely beyond the scope of any individual study or programme of research. Further, these models lack direction for how to achieve cohesiveness toward a common goal. Additionally, despite a need for evidence-informed policy and practice for health [14, 17], there are few examples of using IKT approaches to respond to wicked problems. These features that constrain the application of IKT in the face of wicked problems could be redressed through adoption of the principles of GHG, particularly its mechanisms of legitimacy and collaboration between multi-sector transnational actors, with an emphasis on civil society.

## What does GHG bring to the table?

As a reaction to the intensifying wickedness of health problems that defy state borders, governance processes consist of stakeholders working through formal international institutions both within and across borders. Heavily influenced by major globalisation events such as HIV/AIDS and SARS, current mechanisms and processes for GHG stem from the disciplines of political science, health economics and health policy [72, 73]. In the absence of a singular global government, GHG platforms convene a plurality of major actors to define shared values, establish standards and regulatory frameworks, set priorities, mobilise and align resources, and promote research. GHG often requires individual governments to forgo aspects of their individual sovereignty in order to collaborate and participate with international agencies such as WHO [74]. For example, the WHO International Health Regulations establishes standards for how individual countries respond to international health risks [75]. These regulations refer to the need for the Director-General of WHO to consider scientific evidence, but do not provide recommendations for how this evidence could inform decision-making.

Ultimately, GHG is a polycentric system that provides a mechanism for collective decision-making for improved health through the interplay of different institutional forms and actors at different levels in pursuit of common goals [29, 73, 76]. The imperfect decision-making processes of GHG are, however, embedded in historical and socio-political contexts of colonialism and heavily influenced by power relationships, values, norms, organisational structures and resources. GHG is political; it can serve to reinforce or challenge existing institutional exclusion and power inequalities and has direct impacts on health system equity whereby the decisions made through GHG processes shape who accesses benefits and whose voices are heard [77]. Continued processes of globalisation and increasing influence of private sector actors in global health bring new layers of political power to the governance scene [78], while innovations in technology, data, communications and networks open possibilities for reimagining the mechanisms and processes relied upon post World War II [79]. The time is ripe for reimagining how GHG might better support collective responses to global problems.

At the turn of the twenty-first century, health sectors worldwide were acutely aware of their limited capacity to deal with emerging challenges in isolation. Global vulnerability to pandemics, climate change and political instability all contribute to a growing recognition of a need for multi-sectorial action and broad public and private partnerships at national and international levels [74]. Further, civil society and political leaders are challenging notions of an isolated, technocratic health sector and call for more unified attention to issues of equity and human rights [73]. Society writ large voiced a desire to be part of the political sphere that shapes their life circumstances, opportunities and experiences of health and healthcare. In response to a confluence of heightened awareness of the globalised nature of health issues and growing demand for collective responses, complex networks of international agencies and philanthropic foundations collaborated to set global targets for progress toward a more equitable word through the Millennium Development Goals and the more recent Sustainable Development Goals [33, 80]. These and other examples of governance for health equity (e.g. the WHO Commission on Social Determinants of Health) are key demonstrations of the kinds of platforms and mechanisms GHG offers. Importantly, these mechanisms also demonstrate how the legacy of colonialism contributes to health equity failures.

Global health crises exemplify how health equity is tied up to socio-political and economic contexts, including the histories of colonisation. The 2014–2016 Ebola epidemic is an important example of the consequences of governance failures. As outbreaks emerged, the world witnessed vulnerabilities and fragmentation in public service sectors that became determinants of who lived and who died – revealing intense inequities between and within countries [81–83]. Leaders in health systems and governments alike recognised the need for strong global institutions, mechanisms and funding for development of global public goods that contribute to resolving global health threats. In the case of the Ebola crisis, GHG leadership (e.g. WHO) failed to respond in a timely manner, which lead to other key actors stepping up to fill the leadership gap. The response was openly criticised as “*too little too late*” to halt an epidemic reflective of the “*pathology of society and the global and political architecture*” [84]. Like many contemporary GHG challenges, this crisis unfolded through the legacy of colonialism [85] that holds the roots of inequities in place. By revealing the differential value placed on human life globally, these failures illuminated the tenacious nature of health inequities and the lack of political will to uproot their causes.

While GHG provides a platform for responding to wicked problems through global collaboration, cooperation and leadership among a diverse set of actors, GHG deliverables still lack strategies to ensure evidence- [86] and equity-informed [87] policy, practice and decision-making. The 2014 Lancet–University of Oslo Commission on Global Governance for Health also pushed for evidence- and equity-informed GHG, recommending mandatory health equity impact assessments for all global institutions and strengthened sanctions against non-state actors for rights violations [88]. Surprisingly, there are few examples of looking to IKT to support processes for the same [89]. Shared governance and public dialogue about our social and economic architecture is needed [90], where public moral norms can be re-constructed and internalised (e.g. recreating constructs of health equity as a public good). IKT approaches and strategies could support this kind of dialogue in engaged, inclusive ways that support connecting this kind of evidence and other knowledges with action. In Table 2, we offer an overview of recognized steps in the knowledge-to-action cycle [25] alongside complementary GHG processes and mechanisms. This blended IKT–GHG approach, done alongside a critical examination of power, presents a promising pathway toward health equity action.

**Table 2:** Blending processes and mechanisms for a blended integrated knowledge translation (IKT) – global health governance (GHG) approach

Moments in the IKT cycle	Complementary GHG processes and mechanisms	Examining Power in an IKT–GHG Approach
Identify problem and identify, review, select knowledge ↓ Adapt knowledge to local context ↓	Governance bodies that work together to identify problems and knowledge Consideration of the composition of non-traditional actors, such as civil society and private sector, in governance bodies Guidance for meaningful engagement between actors, particularly in shared governance models Promising example: GAVI mitigates known global power imbalances through the composition of their Board, which includes 9 neutral individuals who speak to public interests, 5 government representatives each from donor and recipient countries, 1 expert in research and technology, 1 industry representative each from the global South and global North, 1 civil society representative, and 1 representative each from WHO, UNICEF, World Bank and Bill & Melinda Gates Foundation	Taking steps to balance power between global North and global South Promoting transparency and accountability in decision-making about the composition of governance bodies Attentiveness to how particular ways of framing health and governance influences how a ‘problem’ is being understood Attentiveness to how historical conditions and power dynamics privilege particular assumptions
Assess barriers to knowledge use ↓ Select, tailor, implement interventions ↓	Guidance on how to resolve discrepant norms and values between engaged actors Guidance on how to ensure legitimacy of leadership Guidance on how political will and power influence this process Platforms for coordinating global-level responses to wicked problems Promising example: The Lancet Commission on GHG offered specific recommendations for governance mechanisms and processes, with detailed calls to make the examination of issues of power an explicit responsibility of GHG. They called for attention to democratic deficit, institutional and structural inflexibility, strengthened accountability, identification and involvement of missing institutions and voices, and to create a policy space for health. Their report offers specific guidance on how to do so. Among the Commission’s recommendations were specific mechanisms, including a proposed UN Multi-stakeholder Platform on Global Governance for Health	Attentiveness to how historical conditions and power dynamics give rise to inequities in inclusion and voice Exploration of how processes of historical exclusion (e.g. due to race, class, gender, Indigeneity, etc.) can be mitigated
Monitor knowledge use ↓ Evaluate outcomes ↓ Sustain knowledge use	Generation and maintenance of mechanisms provide infrastructure for monitoring and evaluation Norms and expectations for transparency in decision-making Promising example: Two advisory bodies, the Technical Review Panel and a Technical Evaluation Reference Group, provide independent audit and monitoring of programmes funded by the GFATM. Their reports highlight lessons learned from funding requests and reviews, including perspectives of applicants, technical partners, the Secretariat and the Board. They consist of external experts in HIV, TB and malaria as well as experts in human rights, gender, health systems and sustainable financing. Their reports are made publicly available through the GFATM website	Attentiveness to who decides what knowledge count as legitimate Attentiveness to who decides what outcomes count as legitimate Consideration of who owns knowledge, with efforts to promote publicly owned and accessible data Attentiveness to equitable distribution of resources and benefits

## Additional ‘objects’ of consideration on this dialogic table

In addition to our interest in leveraging the relational-dialogic nature of GHG and IKT to counter reductionism and mitigate power imbalances, we propose placing a few additional objects on this dialogic table, namely accountability, leadership and inclusion. It is beyond the scope of this discussion to resolve the intricacies of any of these issues, but we hope that they serve as sparks for continued dialogue and reflection. In GHG, the lack of accountability of major global health organisations (i.e. WHO), and its relationship to systems of power, has been a significant challenge [74]. Unclear accountabilities, particularly for leadership, can play a role in legitimising investments in research, IKT, or policy in ways that overlook evidence about causal roots or reinforce inequitable power dynamics. Without frank acknowledgement of the legacies of colonisation, and particularly at a time when neoliberal reason and monetisation of socio-political processes undermine democratic governance [85, 91], it is insufficient to assume health equity is the responsibility of governments, government agencies or civil society, nor of international institutions, such as WHO or United Nations, whose political leverage falls under the shadow of powerful financial bodies such as the World Trade Organization, International Monetary Fund, World Bank and, more recently, influential and well-endowed philanthropic foundations such as the Bill & Melinda Gates Foundation [78, 79]. Neither can the roles, responsibilities and accountabilities of ‘researchers’ and ‘research users’ in IKT be simply assumed because they agree to work together. These are critical considerations in moving toward evidence-informed, equitable governance for health equity action.

Systems for enabling accountability and transparency must be agreed to, which raises questions of meaningful participation and responsibility [30]. Despite intense imbalances in power and interests, the challenge for GHG and IKT strategists alike will be to engage a plurality of actors in ways that enable collective agreement on a common goal. Accountability extends to issues of inclusion and exclusion and how power is distributed. Though inclusion is widely recognised as important for GHG and KT, how to achieve it is elusive. Global events exemplify ways in which civil society is pushing back on systems of exclusion, voicing a desire to transform what are, in essence, governance processes. Responses to global health issues evolve in politicised systems that exclude the voices of those most burdened by health inequities [92]. The Idle-No-More [93], Occupy [94], Black Lives Matter [95] and the more recent #metoo movements share a common outcry for justice and equity in society, pointing to the inequities generated by power and policy structures that systematically privilege the wealthy and White. Collectively, these movements reflect a growing public demand for politics of inclusion where government and non-government actors are held accountable for the consequences of their action (or inaction). They are demonstrative of how intricately tied up health inequities are in complex, competing systems of power within which there is a need for critical analysis and mitigation.

Further, the likelihood of understanding complexity becomes much greater by directly fostering balanced representation that includes a pluralism of voices. On a larger scale, this is reflected in the evolution of the major GHG players in the world. Historically, WHO and the World Bank have been primarily responsible for GHG, but given the significant frustration with each of these institutions’ poor GHG, two new organisations have risen, namely The Global Fund for AIDS, TB, and Malaria (GFATM) and Global Alliance for Vaccines and Immunization (GAVI); what separates these two institutions from their counter parts are their commitment to GHG. These commitments include a wider, more inclusive, Board of Governors (civil society, the private sector, and philanthropic organisations), as well as providing clear and transparent (i.e. publicly available) decision-making regarding funding decisions and priority-setting processes. Both organisations rely on external review for their accountability for decision-making processes like funding decisions. We believe that theory and practice in both IKT and GHG would benefit from these new examples of creating organisations that work toward governance models based on inclusion, voice, transparency and accountability. Without clear leadership and a commitment to accountability through transparency by all global health actors, the current response to health inequities will be ad hoc and exclusive of these civil society voices, as well as highly fragmented with little to no formal mandate between the players. Importantly, the response would be at risk of remaining distracted by the tendency to focus on symptoms rather than causes.

As the field of IKT evolves, so too do opportunities for theory and practice refinement. Governance processes could enhance current IKT frameworks to open considerations of how to weave evidence into decision-making while acknowledging conflicting norms and values within the political sphere under which it operates. Using shared health governance theory to drive this examination can contribute to more transparent and equity-centred approaches to understanding how these norms and values shape health problems [90]. Expanding IKT models to include governance would require re-examining legitimacy, transparency, power and inclusion in the process of connecting knowledge with action*.* This broader conceptualisation extends the application of IKT into a complex public sphere, across domains and outside the control or context of any one institution or set of actors. We are much more likely to approach understanding complexity through systems of inclusion that directly engage multiple socio-political arenas. Systems of inclusion can be explicitly addressed by adopting principles of GHG alongside the strategies of IKT.

Exploring a blended IKT–GHG approach could extend insights from the success of IKT in clinical and health systems settings [70, 96] to wicked problems. This approach could illuminate new ways of thinking about how we might influence the trajectory of wicked problems to fair, equitable governance informed by high quality, rigorous and relevant research. In the example we offer here, of moving toward health equity, IKT implicates an all-of-society approach because the root causes involve all of society. IKT models already acknowledge the process of connecting knowledge with action as inherently social, but this is often used as a way to describe the processes involved in well-defined settings. Wicked problems are not confined to singular contexts. Although attending to social processes are important, they need to be considered in the broader sphere of how society is organised. This means thinking about and connecting the best available knowledge about a wicked problem to evidence-informed action as a ‘public good’, wherein the process is integrated as part of the social fabric around which communities are organised. We believe broadening the application of approaches to IKT across multiple layers of complex social interactions can support evidence-informed influence and, again, GHG can support the achievement of coherency in doing so.

## Conclusion

In this article, we set a dialogic table to explore how blending principles of GHG with IKT strategies could leverage the strengths of both, enhancing the possibility for effective and evidence-informed answers to wicked problems. We situated this table in a global political economy that unfairly distributes power, resources and money. By focusing on explicit examination of power and overcoming mechanistic assumptions that draw attention away from the root causes of health inequities, there is tremendous potential to be leveraged in a combined IKT and GHG approach. Such an approach would require leadership from academic, policy and civil society arenas wherein existing GHG platforms explicitly embrace a commitment to connecting knowledge (evidence about causes) with action. We encourage those pursuing an IKT–GHG approach to engage in bold and inclusive dialogue about how socio-political histories (e.g. colonisation) are at play in the ways they frame or respond to health inequities. In contemplating governance-focused IKT, actors involved in advancing health equity can take promising steps toward inclusion of a broad spectrum of actors and a pathway for stimulating the collective agency needed to affect change on this wicked problem.

## Abbreviations

GHG: global health governance
IKT: integrated knowledge translation
KT: knowledge translation
